# Long-term trends and seasonality of omphalocele during 1996–2010 in China: a retrospective analysis based on the hospital-based birth defects surveillance system

**DOI:** 10.1186/s12884-015-0530-3

**Published:** 2015-04-25

**Authors:** Xiaohong Li, Li Dai, Yanping Wang, Lin Yi, Changfei Deng, Kui Deng, Guangxuan Zhou, Qi Li, Zheng Liu, Ying Deng, Jun Zhu, Xiaosong Li

**Affiliations:** Department of Epidemiology and Health Statistics, West China School of Public Health, Sichuan University, Sec.3 No.17, South RenMin Road, Chengdu, Sichuan 610041 China; National Center for Birth Defect Monitoring, West China Second University Hospital, Sichuan University, Sec.3 No.17, South RenMin Road, Chengdu, Sichuan 610041 China; Laboratory of Molecular Epidemiology for Birth Defects, West China Second University Hospital, Sichuan University, Chengdu, Sichuan China

**Keywords:** Omphalocele, Birth prevalence, Secular trend, Seasonality, Negative binomial cyclical regression model

## Abstract

**Background:**

Little is known about secular trends and seasonal variation in the birth prevalence of omphalocele in China. This study aimed to explore the long-term trends and seasonality of this birth defect, to provide insight into the etiology and prevention of omphalocele.

**Methods:**

A retrospective analysis of all births with omphalocele (1322 cases in 8.8 million births) registered in the hospital-based Chinese Birth Defects Monitoring Network between January 1996 and September 2010. Negative binomial cyclical regression models were used to analyze the long-term trends and seasonal fluctuations of omphalocele occurrence in the southern and northern regions and urban and rural areas of China.

**Results:**

The total prevalence of omphalocele was 1.50 cases (95% confidence interval (CI): 1.42–1.58) per 10,000 births. There was no significant secular trend of omphalocele occurrence in China between 1996 and 2010. The observed prevalence of omphalocele in rural areas was 2.03–2.54 cases per 10,000 births between May and August, which was higher than that observed in other months. The highest prevalence of births with omphalocele in rural areas occurred at the end of June; on average, the prevalence of omphalocele at that time point increased by 20% (95% CI: 6–35%) compared with other months.

**Conclusions:**

There were no long-term trends found for occurrence of omphalocele in China between 1996 and 2010; however, seasonality was observed for omphalocele in women living in rural areas. These results may help generate hypotheses for further study of environmental factors that vary by season.

## Background

Omphalocele is a rare congenital abdominal wall defect in which abdominal organs, such as the intestines, liver, and occasionally other organs, are displaced outside of the abdomen in a translucent sac as a result of anterior abdominal wall dysplasia. Omphalocele has been reported to occur in 0.74–5.13 per 10,000 live births in various countries [1]. Omphalocele is usually associated with severe recognized or unrecognized congenital abnormalities, such as chromosomal abnormalities, cardiac anomalies, and nervous system malformations, and it has a high mortality rate of 15–37% [2-4].

Exposure of pregnant women to certain environmental factors is an important cause of congenital malformations [5]. Several studies suggest that the occurrence of omphalocele is greater in women who live close to landfill sites and in those who take certain prescription medications or excessively use tobacco or alcohol during pregnancy [6-8]. However, there is little clear evidence about what the environmental agents for omphalocele are, thus its causes largely remain an enigma. Previous studies have indicated that an exploration of the seasonality of congenital abnormalities, by identifying seasons with high and low occurrence and narrowing the range of suspicious risk factors, is an effective method to search for the causes of this birth defect [9-11]. Several studies have been conducted to identify the seasonality of omphalocele in the last 30 years; however, the conclusions been inconsistent. Some studies failed to show any significant seasonal variation in the incidence of omphalocele [12,13], whereas seasonality of omphalocele was observed in another study [14]. Additionally, studying secular trends of the birth prevalence of omphalocele is important for targeting effective public health strategies. However, few reports about the occurrence of omphalocele in China have been published, and the prevalence and time trends of this disease in the country during recent years remain unknown.

This study aimed to analyze the seasonal variation and long-term trends in live births with omphalocele using 15 years of consecutive data from the national birth defects registry of China, and to provide insight into the etiology, prevention, and control of this congenital anomaly.

## Methods

### Data source

Data used in our study on the occurrence of omphalocele between January 1996 and September 2010 were retrieved from the national birth defects surveillance database maintained by the hospital-based Chinese Birth Defects Monitoring Network (CBDMN). Although the database we used is not freely available, we have obtained the permission from the the Division of Maternal and Child Health Services, National Health and Family Planning Commission of China, to use it. The CBDMN included approximately 460 hospitals during the period 1996–2005 throughout 116 cities or counties in 31 provinces, municipalities, or autonomous regions of China. Approximately 300 new member hospitals in 220 cities and counties were added to the CBDMN in 2006, but we did not use data from these hospitals in this study because of concerns regarding consistency of the data source and the relatively low data quality. Surveillance subjects in the CBDMN consisted of all live births, stillbirths and terminations of pregnancy for fetal anomalies at 28 weeks’ gestation or more. If the gestational age at birth was unknown, infants with birth weight greater than 1000 g were also included in the monitored subjects [15,16]. The maximal diagnosis time for birth defects was within 7 days after birth. Cases with omphalocele that were born or induced in member hospitals were required to be registered in the CBDMN.

Ethical approval for our study was provided by the Ethics Committee of West China Second University Hospital, Sichuan University (The Granted number: 2010015).

### Data collection

A three-level (county, provincial, and central) surveillance network and clinical expert groups were established to undertake the data collection [16]. In member hospitals of the CBDMN, each neonate (or terminated fetus) was required to be examined immediately after birth by trained health care professionals, to screen for congenital anomalies. Each case of an abnormality required confirmation by experts in the departments of pediatrics or obstetrics or ultrasound experts at member hospitals. Cases in which abnormalities had been confirmed by a prenatal diagnosis were reconfirmed by experts after birth. When the diagnosis of a case was unclear, the staff (usually the nurse) responsible for birth defect monitoring at the hospital collected more details (e.g., medical records, photos of the case) to be used for rediagnosis by the higher-level expert group. For each birth defect case, the staff was responsible for gathering information (e.g., family socioeconomic and demographic information, clinical features, and obstetric items) through interviews with the mothers or medical record reviews. Additionally, the number of maternal age-specific, residential-specific (urban and rural), and sex-specific births were also collected monthly [17]. The data were regularly entered into the online reporting system for maternal and child health (MCH) surveillance (http://zhibao3.mchscn.org) by specialized staff at the county-level MCH hospital.

### Statistical analysis

Cases of omphalocele in this study were diagnosed in accordance with the International Classification of Diseases, Tenth Revision (Q79.2). All isolated, multiple cases of omphalocele were included in our analysis. The prevalence proportion was used to describe the occurrence of omphalocele. This value was expressed as the number of omphalocele cases in newborns at 28 weeks’ gestation or more per 10,000 births (including live births and stillbirths). Three associated factors (residential area, geographic region, and maternal age) were included in further analyses. Residential area was categorized as urban (cities, urbanized areas or neighborhood communities) or rural (villages or countryside), according to the last place the mother had resided for at least the previous 12 months. Region refers to the mother's residence location. In China, areas north of the 35th parallel north were classified as the northern region, and areas to the south comprised the southern region. Maternal age was divided into five age groups: <20 yrs, 20–24 yrs, 25–29 yrs, 30–34 yrs, and ≥35 yrs.

Negative binomial cyclical regression models were used to analyze long-term trends and seasonal fluctuations in the occurrence of omphalocele between January 1996 and September 2010. The negative binomial model was selected because omphalocele is a relatively rare event; 72.4% of the region-, residential-, and age-specific number of cases was 0 in a given month. The basic form of the models is expressed as M1:

M1 $$ \ln \left({\mathrm{d}}_{\mathrm{j}}\right)= \ln \left({\mathrm{N}}_{\mathrm{j}}\right)+{\upalpha}_0+{\upalpha}_1T+{\displaystyle \sum_0^k}\psi \cos \left(2k\pi \omega {t}_j-{\uptheta}_k\right)+{\upbeta}_1{\mathrm{X}}_1+{\upbeta}_2{\mathrm{X}}_2+\dots +{\upbeta}_{\mathrm{p}}{\mathrm{X}}_{\mathrm{p}} $$

where j is the time period (j = 1,2,…,j), d_j_ is the number of omphalocele cases in the period j, N_j_ is the number of births in period j, and α_0_ is the logarithm of the baseline hazard function. *T* (year) is the long-term trend of omphalocele occurrence. Seasonal fluctuation is expressed as ${\displaystyle {\sum}_0^{\mathrm{k}}}\uppsi \cos \left(2{\mathrm{k}\uppi \upomega \mathrm{t}}_{\mathrm{j}}-{\uptheta}_{\mathrm{k}}\right)$; *ψ* is the amplitude of periodic fluctuation; *k* is the order of seasonal fluctuation and *k*. usually set as 0, 1, and 2 [18]. If *k* is zero, this implies no seasonal fluctuation in omphalocele. Therefore, *k* is the number of peaks in occurrence of omphalocele in 1 year, and θ_*k*_ is the position of the peaks. *ω* is the length of cycle. In our analysis, we set 12 months in 1 year as equal to a cycle, so ω = 1/12. *t*_*j*_ is the seasonal variable: month. Χ_p_ (p = 1,2,…) represents the risk factors. In our analysis, geographic region, residential area, and maternal age were added to the models as risk factors. Thus, the ratio of omphalocele birth prevalence between the southern and northern regions, adjusted for residential area and maternal age, can be calculated by e^β^.

To facilitate parameter estimation of the model, we transformed *ψ* cos(2*πωt*_*j*_ − θ) into a linear form [19]. We set *ψ* cos(θ) = γ_1_, *ψ* sin(θ) = γ_2_; therefore, *ψ* cos(2*πωt*_*j*_ − θ) = γ_1_ cos(2*πωt*_*j*_) + γ_2_ sin(2*πωt*_*j*_). The parameters *ψ* and θ can be estimated by formula F1:

F1 $$ \left\{\begin{array}{l}\psi =\sqrt{\upgamma_1^2+{\upgamma}_1^2}\\ {}\theta =\left\{\begin{array}{l}{\mathrm{tg}}^{\hbox{-} 1}\;\left({\upgamma}_2/{\upgamma}_1\right)\kern4em {\upgamma}_1>0,\kern1em {\upgamma}_2>0\\ {}\uppi + {\mathrm{tg}}^{\hbox{-} 1}\;\left({\upgamma}_2/{\upgamma}_1\right)\kern3em {\upgamma}_1<0\\ {}2\uppi +{\mathrm{tg}}^{\hbox{-} 1}\left({\upgamma}_2/{\upgamma}_1\right)\kern2.5em {\upgamma}_1>0,\kern1em {\upgamma}_2<0\end{array}\right.\end{array}\right. $$

We set cos(2*kπωt*_*j*_)=c_*k*_, sin(2*kπωt*_*j*_)=s_*k*_*.* Therefore, model M1 is equal to model M2, which was used for the final analysis in our study.

M2 $$ \ln \left({\mathrm{d}}_{\mathrm{j}}\right)= \ln \left({\mathrm{N}}_{\mathrm{j}}\right)+{\upalpha}_0+{\upalpha}_1T+{\displaystyle \sum_0^k}\left({\upgamma}_{\mathrm{k}1}{c}_k+{\upgamma}_{\mathrm{k}2}{\mathrm{s}}_{\mathrm{k}}\right)+{\upbeta}_1{\mathrm{X}}_1+{\upbeta}_2{\mathrm{X}}_2+\dots +{\upbeta}_{\mathrm{p}}{\mathrm{X}}_{\mathrm{p}} $$

We used three models (*k* set to 0, 1, and 2, respectively) to estimate the long-term trends and seasonal fluctuations of omphalocele nationwide, in the northern region, southern region, urban areas, and rural areas. The likelihood ratio test statistic G^2^ was used to explore the significant seasonal fluctuations.

All statistical analyses in this study were performed using SAS 9.3 software (SAS Institute Inc., Cary, NC, USA). The statistical significance level for α was set at 0.05.

## Results

Between January 1996 and September 2010, a total of 1322 omphalocele cases were identified, which yielded a total prevalence of 1.50 cases (95% confidence interval (CI): 1.42–1.58) per 10,000 births. Table 1 shows the prevalence of omphalocele in each year from 1996 to 2010. Total omphalocele prevalence in the southern region was 1.67 cases per 10,000 births in the last 15 years, which was 1.28-fold (95% CI: 1.15–1.43) higher than that in the northern region, after adjusting for maternal age and residential area. The omphalocele prevalence was 1.81 and 1.35 cases per 10,000 births, respectively, in rural and urban areas between 1996 and 2010.

**Table 1:** **Geographic, urban- and rural-specific birth prevalence of omphalocele (per 10,000 births) in China, 1996–2010**

**Year**	**South**		**North**		**Urban**		**Rural**		**Total**	
	**Cases**	**Prevalence(95% CI)**	**Cases**	**Prevalence(95% CI)**	**Cases**	**Prevalence(95% CI)**	**Cases**	**Prevalence(95% CI)**	**Cases**	**Prevalence(95% CI)**
1996	31	1.44(1.42-1.47)	25	1.21(1.19-1.25)	36	1.21(1.20-1.24)	20	1.59(1.56-1.65)	56	1.33(1.32-1.34)
1997	30	1.50(1.48-1.53)	16	0.73(0.71-0.77)	32	1.08(1.07-1.11)	14	1.14(1.10-1.20)	46	1.10(1.09-1.12)
1998	42	2.03(2.01-2.06)	29	1.30(1.29-1.34)	42	1.39(1.38-1.41)	29	2.28(2.25-2.34)	71	1.65(1.64-1.67)
1999	42	1.90(1.88-1.93)	41	1.82(1.80-1.85)	53	1.69(1.68-1.71)	30	2.25(2.22-2.31)	83	1.86(1.85-1.87)
2000	44	1.77(1.75-1.80)	39	1.55(1.53-1.57)	55	1.56(1.54-1.58)	28	1.90(1.87-1.95)	83	1.66(1.65-1.67)
2001	37	1.54(1.53-1.57)	30	1.24(1.22-1.27)	45	1.37(1.36-1.39)	22	1.42(1.40-1.47)	67	1.39(1.38-1.40)
2002	34	1.31(1.30-1.34)	41	1.54(1.52-1.56)	44	1.22(1.21-1.24)	31	1.87(1.84-1.91)	75	1.43(1.42-1.44)
2003	36	1.46(1.45-1.49)	30	1.55(1.53-1.59)	37	1.27(1.26-1.30)	29	1.93(1.91-1.98)	66	1.50(1.49-1.52)
2004	54	1.71(1.70-1.73)	47	1.58(1.56-1.60)	68	1.61(1.61-1.63)	33	1.70(1.68-1.74)	101	1.64(1.64-1.65)
2005	55	1.71(1.69-1.73)	33	1.15(1.14-1.18)	47	1.15(1.14-1.17)	41	2.05(2.03-2.09)	88	1.45(1.44-1.46)
2006	48	1.39(1.38-1.41)	49	1.55(1.53-1.57)	61	1.40(1.39-1.41)	36	1.59(1.57-1.62)	97	1.46(1.46-1.47)
2007	69	1.63(1.62-1.64)	51	1.28(1.27-1.30)	70	1.26(1.25-1.27)	50	1.89(1.88-1.92)	120	1.46(1.46-1.47)
2008	81	1.83(1.82-1.84)	52	1.30(1.29-1.31)	72	1.30(1.29-1.31)	61	2.10(2.08-2.12)	133	1.57(1.57-1.58)
2009	93	2.00(2.00-2.02)	52	1.23(1.22-1.24)	82	1.46(1.46-1.48)	63	1.92(1.91-1.94)	145	1.63(1.63-1.64)
2010	59	1.58(1.57-1.60)	32	0.96(0.94-0.98)	56	1.22(1.21-1.24)	35	1.40(1.38-1.43)	91	1.28(1.28-1.29)

*Abbreviation: CI* Confidence interval.

**Figure 1:**
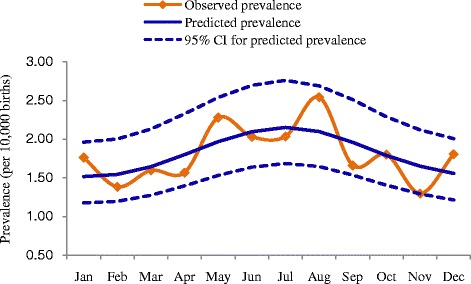
Seasonality of omphalocele in rural China.

**Figure 2:**
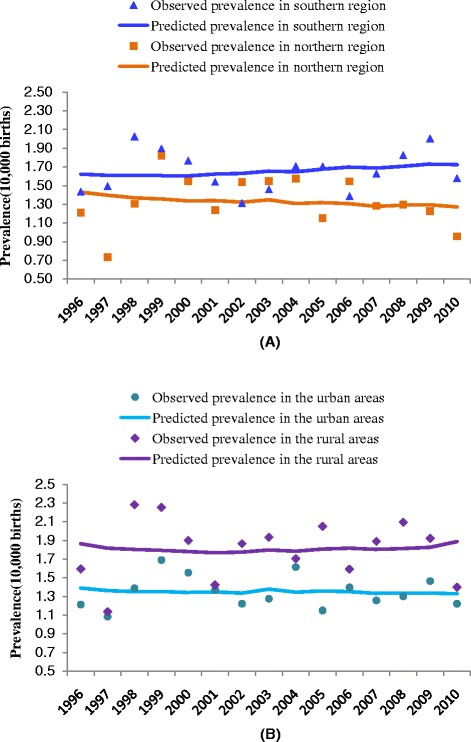
Long-term trends of omphalocele prevalence in northern and southern regions **(A)** and urban and rural areas of China **(B)**.

Table 2 shows a comparison of the fitted results of the three models in each region or area of China, and nationwide. Significant seasonal fluctuations in the occurrence of omphalocele were observed in rural areas. The prevalence of omphalocele in rural areas was 2.03–2.54 cases per 10,000 births between May and August, which was higher than the prevalence in other months (see Figure 1). The results of the model showed that the time point with the highest birth prevalence of omphalocele occurred at the end of June (θ =3.593); this omphalocele prevalence was an average of 20% higher ((e^ψ^ − 1)*100%; 95% CI: 6–35%) than in other months. However, seasonal variation in omphalocele prevalence was not observed in urban areas or in either the southern or northern geographic regions of China. Figure 2 shows predicted omphalocele prevalence in southern/northern regions and urban/rural areas from 1996 to 2010, estimated by models B1, C1, D1, and E2, respectively. There were no significant long-term upward or downward trends in the occurrence of omphalocele in any of the regions or areas. Results of the parameter estimation for long-term trends are presented in Table 3.

**Table 2:** **Comparison of the fitted results of the three models in each region/area of China**

**Model**	***k***		**Variables**	**Log likelihood**	**G** ^**2**^	***p*** **-value**
National wide	0	Model A1	Region urban–rural age year	−2080.89		
	1	Model A2	Region urban–rural age year c1 s1	−2078.47	4.85^a^	0.088
	2	Model A3	Urban–rural age year c1 s1 c2 s2	−2077.84	1.26^b^	0.533
South	0	Model B1	Urban–rural age year	−1075.90		
	1	Model B2	Urban–rural age year c1 s1	−1075.26	1.27^c^	0.530
	2	Model B3	Urban–rural age year c1 s1 c2 s2	−1074.86	0.81^d^	0.667
North	0	Model C1	Urban–rural age year	−996.45		
	1	Model C2	Urban–rural age year c1 s1	−994.30	4.31^e^	0.116
	2	Model C3	Urban–rural age year c1 s1 c2 s2	−993.52	1.55^f^	0.461
Urban	0	Model D1	Region age year	−1113.58		
	1	Model D2	Region age year c1 s1	−1113.45	0.27^g^	0.874
	2	Model D3	Region age year c1 s1 c2 s2	−1112.17	2.56^h^	0.278
Rural	0	Model E1	Region age year	−964.87		
	1	Model E2	Region age year c1 s1	−960.71	8.31^i^	0.016
	2	Model E3	Region age year c1 s1 c2 s2	−959.90	1.62^j^	0.445

^a^Compared model A2 with model A1; ^b^Compared model A3 with model A2; ^c^Compared model B2 with model B1; ^d^Compared model B3 with model B2; ^e^Compared model C2 with model C1; ^f^Compared model C3 with model C2; ^g^Compared model D2 with model D1; ^h^Compared model D3 with model D2; ^i^Compared model E2 with model E1; ^j^Compared model E3 with model E2.

**Table 3:** **Parameter estimation for secular trends and seasonality of omphalocele in each region/residential area of China**

**Model**	**Variable**	**Estimate**	**Standard error**	**Wald 95% confidence limits**		**Wald chi-square**	***p*** **-value**
ModelA1	Year	−0.005	0.007	−0.018	0.008	0.630	0.427
ModelB1	Year	−0.001	0.009	−0.018	0.017	0.010	0.935
ModelC1	Year	−0.010	0.010	−0.030	0.009	1.100	0.295
ModelD1	Year	−0.007	0.008	−0.024	0.009	0.730	0.393
ModelE2	Year	−0.004	0.011	−0.025	0.016	0.160	0.690
	c1	−0.161	0.063	−0.283	−0.038	6.620	0.010
	s1	−0.078	0.062	−0.200	0.045	1.550	0.214
	ψ	0.179	0.063	0.056	0.302	8.121	0.004
	θ	3.593	0.154	3.291	3.895	545.316	<0.001

## Discussion

Overall, no significant long-term trends could be demonstrated for the entire period in the southern or northern regions of China or in rural or urban areas. Comparing with other countries, the birth prevalence of omphalocele in Japan increased from 0.97 cases per 10,000 births in 1974–1979 to 3.94 cases per 10,000 births in 2005–2009. Such an increasing trend was also observed in Austria (from 1.10 cases per 10,000 births in 1974–1979 to 4.20 cases per 10,000 births in 2005–2009) and North America (from 1.88 cases per 10,000 births in 1980–1984 to 4.00 cases per 10,000 births in 2005–2009) [1]. A retrospective study in northern Germany found that the incidence of fetal omphalocele had remained relatively stable from 1993 to 2007 [13]. An analysis of surveillance data from the New York Congenital Malformation Registry showed that omphalocele prevalence in that state declined during 1992–1999. Our study found that the birth prevalence of omphalocele in China fluctuated between 1.10–1.86 cases per 10,000 births between 1996 and 2010, with no significant change during this period. The average point prevalence of omphalocele during this period was 1.50 cases per 10,000 births; this was much lower than those found for the United Kingdom (5.13), the city of Atlanta in the United States (4.01), India (2.80) and the Czech Republic (2.36), but higher than those found for Israel (0.93) and Mexico (0.74) [1]. In addition to differences in the omphalocele incidence of China compared with other countries, differences in the surveillance methods used by the CBDMN compared with those used for birth defects registries in other countries may be a reason for the difference of omphalocele prevalence in China. A criterion used in most birth defects registries to define stillbirths is gestational age of 20, 22, or 24 weeks; however, 28 gestational weeks is used in the CBDMN. Owing to the development of prenatal screening and diagnosis in China [20], during our study period there may be an increased probability of therapeutic labor induction before 28 gestational weeks among pregnant women whose fetuses were prenatally diagnosed with omphalocele. However, such cases of fetal omphalocele induced at less than 28 weeks were not registered in the CBDMN. Therefore, even if the omphalocele birth prevalence at 28 gestational weeks or more exhibited no change during 1996–2010, the birth prevalence might in fact be increasing if fetal omphalocele cases at less than 28 gestational weeks were included. Additionally, residential and geographic variations in the birth prevalence of omphalocele in China were observed. Similar findings showed a higher risk of omphalocele in residents of rural New York than those living in urban areas of the state [21], and another study showed large geographic variations in Europe [22].

Recent studies suggest that many types of congenital abnormalities display seasonal variation. For instance, birth time peaks of microtia occur in autumn and winter [23], anencephaly has a birth peak in March to August [24], pulmonary valve abnormalities peak in September [24], and congenital cataracts have a conception peak in April [9]. One retrospective study reported the seasonality of omphalocele with peak season from July to December in the state of Washington [14]; however, Hornemann et al. [13] found no difference in the seasonal incidence of omphalocele between summer (April to September) and winter (January to March, October to December) in northern Germany. Our study showed that there was seasonal variation in births with omphalocele in rural areas of China, but we found no such seasonality in urban areas. The higher birth prevalence of omphalocele in rural areas was observed from May to August with a peak at the end of June. The higher number of births with omphalocele in summer, especially from June to July, suggests that women living in rural areas are exposed to one or more environmental risk factors for the disease, the effect of which is exerted in autumn and winter months (October to December); the average gestational age of omphalocele cases was approximately 35 weeks in our study, and the development of this birth defect most likely occurs in the first trimester [25]. It may be worthwhile to investigate an association between omphalocele and maternal exposure to agricultural chemicals, given the annual peak in concentrations of estrogenic endocrine-disrupting compounds that coincide with nutrient enrichment on livestock farms and croplands during the autumn and winter months (the dry seasons) [26]. Additionally, we speculate that there might be some interaction effects of environmental risk factors and urban/rural factors on the occurrence of omphalocele. However, we currently have no further evidence to support this.

### Strengths and limitations

The hospital-based CBDMN in China has relatively representative data, with consistent ascertainment methods, wide geographic coverage and a large sample size. Our study is effective in obtaining accurate birth prevalence-related data and assessing the secular trends and seasonality of omphalocele. However, the study has some limitations. First, data from the CBDMN can only support study of the seasonal variation in omphalocele birth prevalence; the conceptional month-specific incidence of omphalocele cannot be obtained because specific gestational ages are registered in the CBDMN only for births with congenital malformations. Therefore, we can only speculate the probable season of the start of omphalocele occurrence. Second, supposing that there is seasonal variation in the probability of therapeutic induction of labor before 28 gestational weeks, our results for seasonality with respect to omphalocele birth prevalence are inaccurate. However, no evidence currently shows that therapeutic induction of labor for fetal omphalocele varies at different times of the year. Additionally, no significant differences were observed in the distribution of gestational age of omphalocele cases among the different months. In our study, the youngest average gestational age was 35.32 weeks in July, and the oldest average age was 36.37 weeks in August.

## Conclusions

In conclusion, there was no significant secular trend found for the birth prevalence of omphalocele in China, but there was possible seasonality in the prevalence of omphalocele in rural areas. These results may help create prevention strategies for congenital abnormalities and generate hypotheses about environmental factors that vary by season, for future studies.

## Consent

Actually an oral informed consent was obtained from the parents for each case in the process of data collection in CBDMN. CBDMN has been included in the official system of the National Bureau of Statistics of China. According to the Law of Mother and Infant Health, and the Statistics Law of the People's Republic of China, medical staffs in the member hospitals are required to collect and report data on birth defects, and the guardians have an obligation to cooperate with them without the need to sign written informed consent individually.

## Abbreviations

CBDMN: Chinese Birth Defects Monitoring Network
MCH: Maternal and child health
CI: Confidence Interval
